# Role of Cholecystectomy in Symptomatic Hyperkinetic Gallbladder Patients

**DOI:** 10.1155/2021/5569850

**Published:** 2021-03-25

**Authors:** Jai P. Singh

**Affiliations:** Department of Surgery, Oswego Hospital, New York 13126, USA

## Abstract

**Introduction:**

Biliary dyskinesia is defined by a gallbladder ejection fraction (EF) of less than 35% on HIDA scan, and these patients have shown a good response to cholecystectomy. Management of patients with biliary colic symptoms who have a hyperkinetic gallbladder (EF > 80%) is not clearly defined. Herein, I report three cases of the symptomatic hyperkinetic gallbladder that were successfully managed with cholecystectomy. *Case Report.* Patient 1was a 56-year-old female presented with pain in the right upper abdomen for one month. Her workup was unremarkable except for the gallbladder EF of 86%. Patient 2 was a 33-year-old female with similar symptoms and workup with gallbladder EF of 97%. Patient 3 was a 20-year-old female with right upper abdominal pain and gallbladder EF of 91%. Patients 1 and 3 had the normal US, normal CT scan, and normal EGD. Patient 2 had normal US and CT but did not undergo EGD. All three patients underwent laparoscopic cholecystectomy and had complete resolution of their symptoms.

**Conclusion:**

The hyperkinetic gallbladder is a rare phenomenon, which can cause debilitating right upper quadrant pain. All three patients had an excellent response to cholecystectomy. Therefore, it is concluded that the patients with biliary colic and gallbladder EF of 80% or higher should be strongly considered for surgery.

## 1. Introduction

Biliary dyskinesia is a common gallbladder functional disorder defined by an ejection fraction (EF) of less than 35% on cholecystokinin 99m technetium-labeled hepatoiminodiacetic acid (CCK-HIDA) scan. Studies have shown a good symptomatic response to cholecystectomy in these patients [1–5]. Management of patients with biliary colic symptoms who have high gallbladder ejection fraction is not clearly defined. A gallbladder ejection fraction of 80% or greater is commonly used to define hyperkinetic gallbladder [6–9]. The hyperkinetic gallbladder is believed to be secondary to rapid contraction and emptying of the gallbladder. Herein, I present three patients with symptomatic hyperkinetic gallbladder who had an excellent response to laparoscopic cholecystectomy.

## 2. Case Reports

### 2.1. Patient 1

A 56-year-old female presented with intermittent episodes of pain in the right upper abdomen and epigastrium for one month. The pain would get worse after eating food without any association with a specific kind. She complained of nausea as well. Her clinical exam was unremarkable. The patient had an extensive workup for her pain, which included abdominal ultrasound (US), computed tomography (CT) scan, esophagogastroscopy (EGD), and colonoscopy and labs. Her workup was essentially normal except for the cholecystokinin 99m technetium-labeled hepatoiminodiacetic acid (CCK-HIDA) scan finding of gallbladder ejection fraction of 86%. The patient did complain of pain at the time of the HIDA scan. Diagnosis of the hyperkinetic gallbladder was made, and laparoscopic cholecystectomy was performed. The patient had slight omental adhesions to the gallbladder. Her pathology revealed features of chronic cholecystitis without any evidence of cholesterosis or cholelithiasis. The patient was seen in the office after eight days and again after one month. The patient responded very well to the treatment and had a complete resolution of her symptoms. The patient was also contacted after six months, and she remained symptom free.

### 2.2. Patient 2

A 33-year-old female had pain in the right upper abdomen for around four months. The pain would radiate to the epigastrium and get worse after eating food, especially fat-rich food. The physical exam did not reveal any abnormal findings. She had labs, abdominal US, and CT scan, which were all normal. However, her HIDA scan revealed a gallbladder EF of 97% without any increase in pain at the time of the HIDA scan. She underwent laparoscopic cholecystectomy, which showed a grossly normal gallbladder without any adhesions. Pathology revealed findings of chronic cholecystitis. She had complete resolution of her pain and remained symptom free when she was contacted for long-term follow-up after six months.

### 2.3. Patient 3

A 20-year-old female presented with right upper abdominal pain without any radiation for six months. Her pain was not aggravated by any food. She had labs, abdominal US and CT scan, and EGD, which were all normal. She had a gallbladder EF of 91% on the HIDA scan with no onset or aggravation of her pain during the scan. She underwent laparoscopic cholecystectomy, which revealed a grossly normal gallbladder. The pathology revealed features of chronic cholecystitis. The patient had complete resolution of her symptoms. She remained symptom free when contacted six months later for follow-up.

## 3. Discussion

Cholecystokinin (CCK) is a peptide hormone synthesized by enteroendocrine cells of the duodenal mucosa and secreted in response to fat and protein ingestion [10]. CCK causes contraction of the gallbladder and relaxation of the sphincter of Oddi, facilitating the release of bile into the intestine [11, 12]. It also causes increased secretion of hepatic bile and pancreatic enzymes [13, 14]. The pathophysiology of abdominal pain due to gallbladder hyperkinesia is not very clear. One possible explanation could be the increased density of cholecystokinin receptors and/or increased secretion of CCK in patients with a hyperkinetic gallbladder, which would cause intense contraction of the gallbladder in response to the ingestion of fat and protein, resulting in abdominal pain. Intense contraction and rapid emptying of the gallbladder could also lead to increased intraluminal pressure that could cause mucosal injury and chronic inflammation, as seen on histopathology [15, 16]. In the current case series, all patients had chronic cholecystitis on their pathological analysis. Similar findings were reported by Saurabh and Green, who found the presence of chronic cholecystitis on pathology in 90% of patients (*n* = 32) with the hyperkinetic gallbladder [15]. Lindholm et al. reported chronic cholecystitis in 100% of patients (*n* = 12) with the hyperkinetic gallbladder [6].

All three patients in this case series had complete resolution of symptoms after cholecystectomy. These findings were consistent with the results reported by Saurabh and Green, who reported symptomatic improvement in 90% of patients, while Lindholm et al. reported complete resolution of symptoms in 100% of patients [6, 15]. Saurabh and Green reported full resolution of symptoms in 74% of patients, while 16% showed partial relief of symptoms after cholecystectomy [15]. Similarly, Holes-Lewis et al. reported symptomatic improvement in 97% of patients with complete resolution of symptoms in 79% of hyperkinetic gallbladder patients after cholecystectomy [8].

CCK levels were not measured in these patients or any other case series related to gallbladder hyperkinesia. Future studies looking into CCK levels might provide more insight into the pathophysiology of gallbladder hyperkinesia.

In a study performed by DuCoin et al., almost 90% of patients with biliary colic, gallbladder EF > 35%, and symptoms reproducible with CCK injections had a complete resolution of symptoms with cholecystectomy [17]. Another study by Morris-Stiff showed that reproduction of symptoms with CCK injection was superior to ejection fraction measurement in predicting a resolution of symptoms after cholecystectomy in patients with biliary dyskinesia [18]. The reproduction of symptoms with CCK injection could be a very reliable indicator for cholecystectomy; however, in the current case series, only one patient reproduced symptoms by CCK injection, while all three patients had complete resolution of symptoms after cholecystectomy. Therefore, patients who do not reproduce symptoms and have high ejection fraction should also be considered for cholecystectomy as the current case series showed complete resolution of symptoms in all three patients. Similar findings were reported by Saurabh et al., who noted that out of 90% of the hyperkinetic gallbladder patients who had improvement in symptoms after cholecystectomy, only 61% had a reproduction of symptoms with CCK injection [15].

In this case series, no correlation was found between intraoperative findings and resolution of symptoms after cholecystectomy. Out of three patients, only one patient had omental adhesions, while two patients had normal-looking gallbladder. Similarly, Saurabh et al. found that 29% of patients had normal-looking gallbladder in the complete response group. In contrast, in the no-response group, all patients had omental adhesions to the gallbladder's anterior surface [15]. This highlights the point that the hyperkinetic gallbladder is a functional problem of the gallbladder and not anatomical.

In the current series, all patients were contacted six months after their surgery to confirm that they remained symptom free even after a long time. This series's limitation is the small sample size; however, the hyperkinetic gallbladder is a rare entity, and there is only limited literature available, and most of the published data have a small sample size.

## 4. Conclusion

In this case series, all patients with biliary colic-like symptoms and hyperkinetic gallbladder on HIDA scan had complete resolution of their symptoms after cholecystectomy. Therefore, the case series concluded that patients with biliary colic and gallbladder ejection fraction of 80% or higher should be strongly considered for cholecystectomy.
